# *ATHB1* Interacts with Hormone-Related Gene Regulatory Networks Involved in Biotic and Abiotic Stress Responses in *Arabidopsis*

**DOI:** 10.3390/cells14181456

**Published:** 2025-09-17

**Authors:** Valentina Forte, Sabrina Lucchetti, Andrea Ciolfi, Barbara Felici, Marco Possenti, Fabio D’Orso, Giorgio Morelli, Simona Baima

**Affiliations:** 1 Council for Agricultural Research and Economics, Research Centre for Genomics and Bioinformatics, Via Ardeatina 546, 00178 Rome, Italy; valentina.forte@crea.gov.it (V.F.); andrea.ciolfi@opbg.net (A.C.); barbara.felici@crea.gov.it (B.F.); marco.possenti@crea.gov.it (M.P.); fabio.dorso@crea.gov.it (F.D.); giorgio.morelli.crea@gmail.com (G.M.); 2 Council for Agricultural Research and Economics, Research Centre for Food and Nutrition, Via Ardeatina 546, 00178 Rome, Italy; sabrina.lucchetti@crea.gov.it; 3 Accademia Nazionale dei Lincei, 00165 Rome, Italy

**Keywords:** HD-Zip transcription factor, *ATHB1*, ethylene, jasmonic acid, salicylic acid, abiotic stress

## Abstract

ATHB1, an *Arabidopsis thaliana* homeodomain-leucine zipper (HD-Zip) transcription factor, is involved in the control of leaf development and hypocotyl elongation under short-day conditions. As growth adaptation to environmental conditions is essential for plant resilience, we investigated the role of *ATHB1* in the interaction between transcriptional regulatory networks and hormone signaling pathways. We found that wounding, flooding and ethylene induce *ATHB1* expression. In addition, we found that the ethylene signal transduction pathway is also involved in an age-dependent *ATHB1* expression increase in leaves. Conversely, methyl jasmonate (MeJA) application decreases the *ATHB1* transcript level. By exploiting mutant and over-expressing (OE) lines, we also found that the ATHB1 level influences plant sensitivity to the inhibitory effect of MeJA treatment on growth. To gain deeper insights into the regulatory pathways affected by *ATHB1*, we performed a microarray analysis comparing the transcriptome of wild-type and *athb1* mutant plants following exposure to MeJA. Remarkably, although the response to the MeJA treatment was not impaired in *athb1*, several genes involved in jasmonate and salycilic acid signaling were already downregulated in *athb1* seedlings under normal conditions compared to the wild type. Thus, our study suggests that *ATHB1* may integrate different hormone signaling pathways to influence plant growth under various stress conditions.

## 1. Introduction

Plants have evolved intricate adaptive mechanisms to survive biotic and abiotic stresses, activating defense responses through hormone signaling and transcriptional networks. Despite these responses, stress conditions frequently diminish crop yields, presenting economic challenges. With most of the cultivated land worldwide experiencing a single or a combination of stresses, exacerbated by climate change, there is a critical need for resilient crop varieties capable of maintaining high productivity while conserving natural resources. This necessitates extensive research to improve our understanding of plant stress responses and advance genetic technologies for developing resilient varieties. Plant stress tolerance relies on molecular and biochemical adjustments, enabling cells to repair stress-induced damage, accumulate protective molecules, and restore cellular balance. Additionally, triggering developmental changes, such as lateral root and root hair growth during drought, can enhance resource utilization and mitigate stress impacts on the plant. The stress-induced changes in plants can occur in various cellular compartments and are regulated across multiple levels, including perception, signal transduction, transcriptional, post-transcriptional, translational, and post-translational regulation [1].

Hormones play a crucial role in signal transduction and integrating developmental and environmental cues, with some responses being common across different stresses, while others are stimulus-specific [2,3]. Abscisic acid (ABA) is the key regulator in abiotic stress conditions like drought, high salinity, and extreme temperatures. On the other hand, ethylene, jasmonic acid (JA) and salicylic acid (SA) are primarily induced by pathogen attack and wounding, and they are mainly involved in biotic stress responses. Extensive research has demonstrated intricate interactions among these hormones, with both synergistic and antagonistic effects. For example, ethylene interacts with JA and ABA signaling in a cooperative manner in plant defense against pathogen attack [2] and antagonistically in stomatal closure [4]. These interactions occur at various levels within hormone signaling pathways, forming complex regulatory networks that integrate external and internal signals for plant protection and acclimation to the environment. This integration involves genetic reprogramming and modulation of the expression of numerous stress-inducible genes by transcription factors (TFs) [5,6]. Several classes of TFs, including AP2/ERF, DREB, HD-ZIP, NAC and WRKY, are particularly responsive to external stimuli and some TFs have been shown to play an important role in biotic and abiotic stress responses.

Transcriptional changes in response to treatments with ABA have been observed for several *Arabidopsis* HD-Zip I genes [7], and there is evidence that members of clades I and II of the HD-Zip I family have roles related to drought stress and ABA signaling in different plant species [8,9,10,11]. In agreement, phenotypic and transcriptomic analysis of transgenic plants have shown that, in most of the assessed cases, over-expression of HD-Zip I proteins improves drought tolerance, likely through the activation/repression of a set of common target genes [12]. Nonetheless, in addition to the positive effects on water stresses, the over-expression of HD-Zip I proteins very often resulted also in undesirable alterations of the shape and growth of the plant, limiting their potential application for crop improvement and suggesting a role for HD-Zip I proteins in specific growth and/or developmental pathways [12,13]. This role has been clearly shown for ATHB1, the founder member of the HD-Zip I family [14]. ATHB1 has been shown to affect cotyledon expansion and leaf palisade cells development when over-expressed in tobacco [15], and to affect leaf development and margin serration in *Arabidopsis* by directly repressing *miR164*, a negative regulator of *CUC2* [16]. Moreover, it has been demonstrated that ATHB1 plays a role in hypocotyl growth under a short-day regime [17]. Furthermore, *ATHB1* expression has been found to be suppressed by drought, ABA, and NaCl in plants cultured in vitro [7], yet conversely induced following severe drought treatment in soil-grown plants at the reproductive stage [12]. In this condition, the ATHB1 OE plants were found to be drought-tolerant compared with their controls, whereas the *athb1-1* mutant plants were more sensitive [12]. Finally, it has been reported that *ATHB1* expression is positively regulated by ethylene in *Arabidopsis* [18], whereas the tomato ortholog HB1 directly regulates the expression of *1-AMINOCYCLOPROPANE-1-CARBOXYLATE OXIDASE* (*ACO*) gene encoding a key enzyme in ethylene biosynthesis [19]. Although these studies demonstrate an effect of environmental conditions on *ATHB1*, its interaction with the transcriptional regulatory and hormonal networks that connect plant development and stress responses remains largely unknown.

To further investigate ATHB1′s function, we analyzed *ATHB1* expression and found that it is induced by flooding, wounding and ethylene. Moreover, the ethylene signaling pathway was shown to contribute to the developmentally regulated increase of *ATHB1* expression observed in leaves during aging. Additionally, we found that MeJA treatment represses *ATHB1* expression while ATHB1 activity increases MeJA sensitivity. In fact, ATHB1 OE and *athb1* mutant plants display an opposite behavior with increased and reduced growth impairment upon MeJA application, respectively. Nonetheless, comparative analysis revealed that the transcriptional response of the *athb1* mutant to MeJA treatment is overall similar to that of wild-type plants. On the contrary, expression of genes involved in JA and SA signaling was observed to be reduced in *athb1* with respect to the wild type in the transcriptome comparison of plants grown under normal conditions. Taken together, our results suggest that ATHB1 might influence plant growth under stressful conditions, interacting with different hormone signaling pathways.

## 2. Materials and Methods

### 2.1. Plant Materials

The ethylene pathway mutants *ein2-1* and *etr1-1* were obtained by the Nottingham Arabidopsis Stock Centre (NASC) and are in the Co-0 background.

The *athb1-3* allele (line GT6141) was retrieved from the CSHL *Ds* gene trap collection [20] and is in the L*er* background. The insertion had been mapped in the third intron, 995 bp downstream of the ATG, using sequencing of the genomic regions flanking the *Ds* transposable element. RT-qPCR analysis indicates a reduction, between two- and five-fold, of *ATHB1* expression and sequencing of the cDNA indicated the production of an aberrant transcript encoding a truncated ATHB1 protein.

The ATHB1 OE is in the WS background and was obtained by cloning into the pBI101 vector (Clontech, Palo Alto, CA, USA) the fragment, obtained by digestion with Eco*RI* and Xma*I* of the total-ATHB1 construct [15], containing the ATHB1 coding sequence preceded by the −343 bp CaMV *35S* promoter and followed by the *rbcS-E9* terminator. *ATHB1* over-expression of about 30-fold was estimated by RT-qPCR.

The *ATHB1*::GUS reporter is in the WS background and was obtained by cloning a 1870 bp fragment of *ATHB1* genomic sequence upstream the translation initiation codon, amplified with ATHB1 5′p (5′-CCCGGGATCCTGCAGCGGAAGAAATACG-3′) and A1C (5′-CGAATTGGATTCCATGGTTGATCAACAG-3′) primers, in frame with the *β*-glucuronidase (GUS) encoding sequence in the pBI101 vector. This sequence corresponds to the *ATHB1* promoter (1500 bp upstream of the transcription start site) and 5′ UTR (370 bp), including the translation regulatory element encoding CPuORF33 [21]. The recombinant plasmid was checked by sequencing to exclude amplification errors.

Standard methods, as described in [22], were used to introduce the binary vectors with the *35S*::*ATHB1* and *ATHB1*::GUS recombinant constructs into *Agrobacterium tumefaciens* strain GV3101 pMP90, for transformation of *Arabidopsis* plants of the WS ecotype by the vacuum infiltration method and for localization of GUS activity in plant tissues.

### 2.2. Growth Conditions

After stratification of the seeds for 3–4 d at 4 °C in the dark, *Arabidopsis thaliana* plants were grown in controlled conditions at 21 °C, under a 16 h light/8 h dark cycle, on GM medium (1× Murashige and Skoog (MS) salts (pH 5.8), 1% sucrose, 0.8% agar) or in soil. For in vitro growth, seeds were surface sterilized as previously described [23].

In order to test *ATHB1* expression response to hormones and abiotic stress, Co-0 wild-type 7-day-old seedlings were treated with MeJA as described below while plants grown on soil for 3 weeks were either incubated for 4 h with 1 ppm of ethylene in a closed environment (bell jar), submerged with water for 1, 2 or 4 h (flooding) or cut and incubated in a humid chamber for 1, 2 or 4 h (wounding). After treatment, all samples were collected and immediately frozen in liquid N_2_ for RNA extraction.

### 2.3. MeJA Treatment

To test the sensitivity to the hormone, sterile seeds were germinated on GM plates to which equal volumes of a MeJA stock (dissolved in ethanol, final concentration 50 and 100 μM; Duchefa Biochemie, Haarlem, The Netherlands) or ethanol (mock) had been added and observed after 20 days of growth. The experiment was repeated twice with two biological replicates each time.

For microarray expression analysis, 7-day-old seedlings grown on nylon mesh (Nytex 03-100/44, Sefar) in GM plates were transferred to fresh solid medium containing 30 μM MeJA or ethanol (mock). Seedlings were collected after 1 h and 4 h of incubation and immediately frozen in liquid nitrogen for total RNA extraction. The experiment was repeated three times.

### 2.4. Gene Expression Analysis

Total RNA was isolated using the RNeasy Plant Mini Kit (QIAGEN, Hilden, Germany) following the manufacturer’s protocol, including an on-column DNAse I digestion step to eliminate genomic DNA contamination. For cDNA synthesis, 1 μg of RNA was reverse transcribed using the QuantiTect Reverse Transcription Kit (QIAGEN, Hilden, Germany). RT-qPCR was carried out using SYBR Green PCR Master Mix (Applied Biosystems, Foster City, CA, USA) on an ABI Prism 7900HT (Applied Biosystems, Foster City, CA, USA), in accordance with the manufacturer’s guidelines. Each reaction (10 μL total volume) was performed in 384-well plates and contained 0.5 μL of 1:5 diluted cDNA and 300 nM of each primer. All assays were run in triplicate. Primers were designed with Primer Express Software v2.0 (Applied Biosystems, Foster City, CA, USA) and, whenever feasible, spanned exon–exon junctions to avoid amplification of genomic DNA. The sequences of all primers used, along with the corresponding gene accession numbers, are provided in Supplementary Table S3. Relative gene expression was calculated using the 2^−ΔΔCt^ method, normalizing to *ACTIN2* (AT3G18780). Gene expression data for mutants and transgenic lines are shown as log2 fold changes relative to wild type, unless stated otherwise. For each biological replicate, the mean value of three technical replicates was used to determine transcript levels. Unless specified differently, all the RT-qPCR results represent the average of three independent biological replicates, and standard deviations (SD) were calculated using the method recommended by [24].

### 2.5. Microarray Analysis

Total RNA, extracted from three independent experimental replicates of WT (L*er*) and *athb1-3* seedlings treated with MeJA (or mock) for 1 h and 4 h as described above, was hybridized to Affymetrix Gene-1-1-ST *Arabidopsis* Array Plate by the NASC Affymetrix Service (https://arabidopsis.info/affy/link_to_iplant.html; accessed on 9 September 2025). For the statistical analysis of the 24 microarray samples, raw signal intensities were normalized using the GCRMA method, which incorporates probe GC content into the Robust Multiarray Analysis algorithm. Quality control of the data was assessed through NUSE and RLE plots. Additionally, to evaluate the similarity of gene expression profiles under different conditions, we performed principal component analysis (PCA) and hierarchical clustering of the normalized datasets. The Limma R-package v2.16.0 [25] was used for statistical analysis. By using linear data model, empirical Bayes-moderated t-statistics were computed for each biological comparison, selecting as significantly differentially expressed only the probes with a *p*-value adjusted with Benjamini–Hochberg false discovery rate (FDR) test lower than 0.05. Differentially expressed probes were annotated to *Arabidopsis* genes using the NetAffx utility available on Affymetrix website. All microarray data of this study were deposited in the NASCArray database (http://bar.utoronto.ca/NASCArrays/index.php?ExpID=572; accessed on 9 September 2025). To validate the array results, gene expression of selected genes was tested by RT-qPCR. GO term enrichment was analyzed with PANTHER Overrepresentation Test (Released 20240226) version 18.0 (https://www.pantherdb.org/; accessed on 9 September 2025) using default parameters (GO Ontology database Released 17 January 2024 https://zenodo.org/records/10536401; accessed on 9 September 2025). Enrichment significance was calculated by means of Fisher’s Exact test and *p*-values were adjusted for multiple testing using Benjamini–Hochberg FDR. Heatmap representation and clustering of differential gene expression were created in R using the Pheatmap package [26]. Promoter sequences (3000 bp upstream of translation start site) of the DEGs were retrieved from the genome of *A. thaliana* using TAIR or RSAT Plant tools (https://rsat.eead.csic.es/plants/retrieve-seq_form.cgi; accessed on 9 September 2025). RSAT Plant was used for scanning the ATHB1 binding site in the promoter regions [27].

## 3. Results

### 3.1. ATHB1 Expression Is Stress Inducible and Regulated by Ethylene

**Figure 1:**
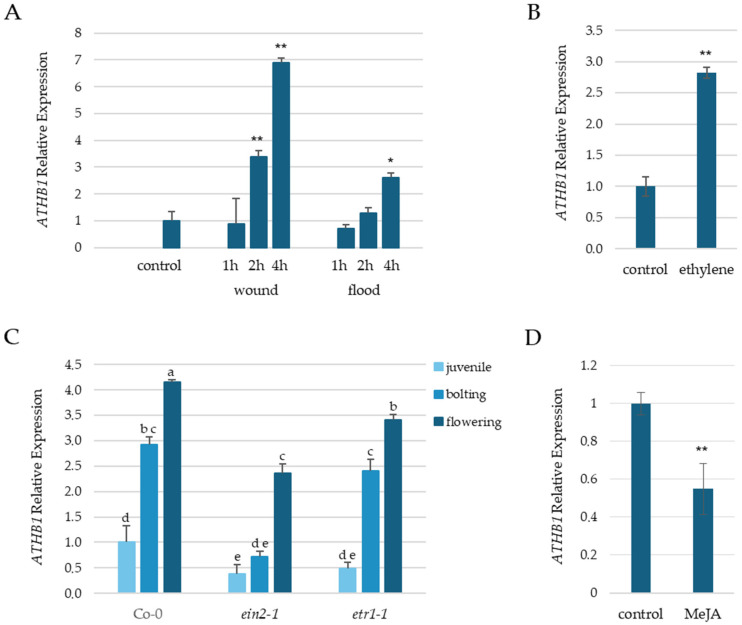
RT-qPCR analysis of *ATHB1* expression. RNA was extracted from: (**A**,**B**) leaves of 3-week-old WT (Co-0) plants submerged (flood) or wounded (wound) for the indicated time or treated 4 h with 1 ppm ethylene; (**C**) 2-week-old seedlings (juvenile) and leaves of WT, *ein2-1* and *etr1-1* plants in two adult stages (bolting and flowering); (**D**) 1-week-old WT seedlings treated 1 h with 30 µM MeJA. Values are mean ± SD (technical triplicates) of relative quantification (2^−ΔΔCt^) with respect to untreated plants (**A**,**B**,**D**) or to 2-week-old WT seedlings (**C**) * *p* < 0.05 ** *p* < 0.01 (Student’s *t*-test). Different letters above the columns indicate statistically significant differences *p* < 0.05 (one-way ANOVA; Tukey test). ACTIN2 was used as reference gene for normalization.

To explore *ATHB1* involvement in the response to adverse environmental conditions, we first analyzed *ATHB1* expression after flooding and wounding of the plants. We found that both treatments increase the *ATHB1* transcript (Figure 1A), suggesting that *ATHB1* could take part in the plant response to these stresses.

By promoter-GUS analysis, we noticed that *ATHB1* expression increased with leaf age (Supplementary Figure S1A), in agreement with expression data reported in the BAR repository of the University of Toronto (https://bar.utoronto.ca; accessed on 9 September 2025) and in [28]. In flowers, GUS staining was observed in the stigma, stamen filaments and in the floral organ abscission zone at the base of developing siliques (Supplementary Figure S1B). It is known that ethylene signaling plays a key role in abiotic and biotic stress responses, leaf development, and in senescence and abscission of vegetative and floral organs [28,29,30]. Intriguingly, we observed that exogenous addition of ethylene increases *ATHB1* mRNA accumulation (Figure 1B), further confirming that this hormone is a positive regulator of *ATHB1* expression [18].

To investigate the link between ATHB1 and ethylene during leaf maturation, we analyzed *ATHB1* expression in ethylene signaling mutants at different phases of growth. A reduction in the steady-state level of *ATHB1* mRNA was clearly observed in both *ein2-1* and *etr1-1* ethylene-insensitive mutants (Figure 1C). Nonetheless, age-dependent accumulation of the *ATHB1* transcript was still observed in the two mutants, indicating that *ATHB1* regulation is controlled by both ethylene and developmental signals.

### 3.2. ATHB1 Enhances Sensitivity to MeJA Treatment

It has been reported that ethylene acts both synergistically and antagonistically with JA signaling to regulate plant defense and growth under unfavorable environmental conditions [4,31,32]; moreover, it has been shown that the expression of several key response genes conferring biotic and abiotic stress tolerance is induced by both ethylene and the JA signaling pathway [4,6,31,32]. The enhancement of JA production by wound, cold, drought, salinity and other stress conditions has profound physiological consequences and serves as a signaling mechanism, particularly for systemic spreading of wound response [33,34,35].

To evaluate a possible interaction between *ATHB1* and JA signaling, we first analyzed *ATHB1* transcript level in WT seedlings exposed to 30 μM MeJA and observed a negative regulation of *ATHB1* expression (Figure 1D). Then, we analyzed the effect of reduced and increased *ATHB1* expression (*athb1-3* mutant and ATHB1 OE plants, Supplementary Figure S2) on plant sensitivity to exogenous application of MeJA during vegetative development.

**Figure 2:**
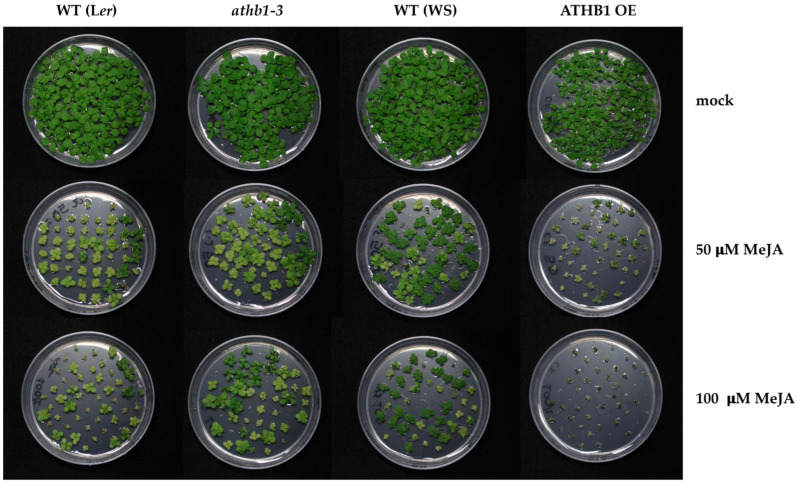
Differential response of ATHB1 OE and *athb1* plants to exogenous MeJA application. WT, *athb1-3* and ATHB1 OE seedlings were grown on plates containing 50 µM or 100 µM MeJA or mock and photographed after 20 days.

When seeds were germinated on plates containing 50 μM or 100 μM MeJA, the growth of wild-type, *athb1-3* and ATHB1 OE plants was impaired in a dose-dependent manner (Figure 2). Nonetheless, while the *athb1-3* mutant plants were less affected, the ATHB1 OE plants were smaller and arrested in their growth, clearly indicating that they are more affected than the parental wild type (Figure 2). This opposite behavior of plants with reduced or increased levels of ATHB1 upon MeJA application suggests that this transcription factor interacts with the JA signaling pathway and influences hormone sensitivity/response downstream of JA.

### 3.3. ATHB1 Affects the Expression of Genes Responsive to MeJA Treatment

To understand how *ATHB1* affects sensitivity to MeJA treatment in *Arabidopsis*, a microarray transcriptomic analysis was performed comparing RNA from 7-day-old WT and *athb1-3* mutant seedlings exposed to MeJA or mock-treated for 1 and 4 h. Surprisingly, only seven genes resulted to be differentially regulated (log_2_FC > 1) with statistical significance (*p*-value < 0.05) in the two genotypes after 4 h of MeJA application, and none after 1 h of treatment (Table 1). Nonetheless, it is noteworthy that all these genes encode regulatory proteins (ZAT10, NAC36, JAZ5, VQ10) or proteins involved in hormone signal transduction (CML38, EDS5, NATA1).

**Figure 3:**
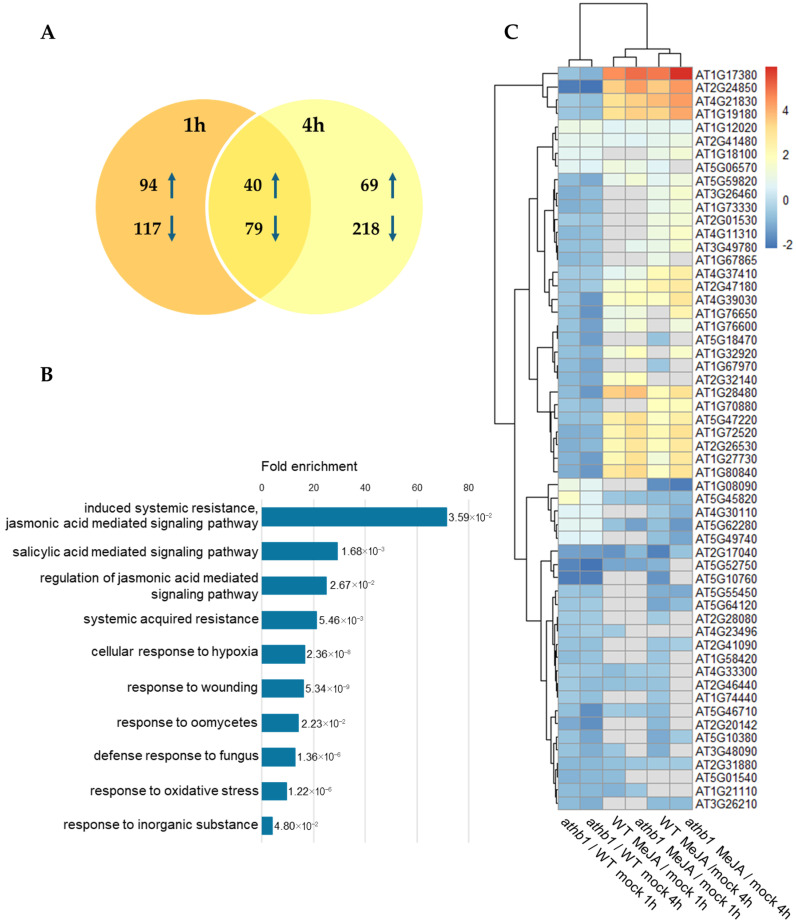
(**A**) Differentially expressed genes (DEGs) between WT and *athb1* mutant. The comparison included triplicate samples of each genotype at two time points (1 and 4 h) after mock treatment. Numbers refer to DEGs, and up and down arrows indicate higher or lower expression, respectively, in *athb1*. (**B**) Gene Ontology (GO) term enrichment of downregulated genes in *athb1* with respect to WT plants. The reported enriched terms represent the most specific subclasses from the same parent term. The length of the bars represents the fold enrichment of the GO term occurrence in the sample with respect to the background (whole genome). The statistical significance (FDR) of the over-representation is indicated at the end of the bar. (**C**) Heatmap depicting the expression patterns of differentially expressed genes in *athb1* with respect to WT-untreated seedlings (*athb1*/WT mock 1 h and 4 h) in response to MeJA application in both genotypes upon 1 h or 4 h treatment. Three biological replicates were used for each time point. Hierarchical clustering and identity of the samples are reported on top and bottom of the columns, respectively. Gene expression clusters are indicated to the right, while AGI codes of genes are on the left. The color scale represents relative expression values (log_2_FC) of microarray analysis, with relatively higher and lower expression levels indicated in orange and blue, respectively. Grey indicates no change upon stimulation.

By looking at the expression of these genes in untreated samples, we observed that almost all were already differentially expressed in the mutant background before MeJA application, while hormone response was similar in the two genotypes. Therefore, we then compared mock-treated WT and *athb1* samples (1 h and 4 h, in triplicate) and found a set of 119 statistically significantly, differentially expressed genes (DEGs, log_2_FC > 0.5), plus *ATHB1* itself (Supplementary Table S1). This gene set is enriched in both abiotic and biotic stress response pathways. Among these genes potentially acting downstream of *ATHB1*, 40 are upregulated in the *athb1* mutant, while the majority (79) are downregulated (Figure 3A), in agreement with the reported transcription activation function of ATHB1 [15].

To gain further information about the genes differentially regulated in *athb1*, the upregulated and downregulated transcripts were separately categorized for gene ontologies (GO). Non-redundant significant over-represented terms in the GO Biological Process (BP) category were identified for downregulated genes (Figure 3B), while no enriched terms were detected for the upregulated ones. The complete list of enriched GO terms is reported in Supplementary Table S2. In agreement with the altered sensitivity to MeJA treatment of *athb1*, the GO terms “induced systemic resistance, jasmonic acid mediated signaling pathway”, “regulation of jasmonic acid mediated signaling pathway” and “systemic acquired resistance” are among the most strongly enriched, together with other GO terms related to “defense response” and “response to biotic stimulus” such as “salicylic acid mediated signaling pathway”, “response to oomycetes” and “defense response to fungus”. In addition, significant enrichment was found for the GO terms “cellular response to hypoxia”, “response to wounding”, “response to oxidative stress”, “response to inorganic substance” and others related to “response to stress” and “response to abiotic stimulus”, thus confirming the interaction of *ATHB1* with the stress signaling regulatory networks.

Interestingly, many of the genes downregulated in *athb1* encode transcription factors. Two of them, *JASMONATE ZIM-DOMAIN 1* (*JAZ1*) and *JAZ5*, encode nuclear transcriptional repressors directly linked to JA response [36]. *WRKY40* and *WRKY70* are involved in integrating the antagonistic effects of JA and SA on defense-related gene expression regulation [37,38,39]. *NAC90* and *NAC36* are members of the *NO APICAL MERISTEM/ARABIDOPSIS TRANSCRIPTION INITIATION FACTOR/CUP-SHAPED COTYLEDON* (*NAC*) family of plant TF that include many regulators of leaf senescence [40]. The *ZAT10* and *ZAT12* genes, encoding C_2_H_2_ zinc finger transcription factors containing an EAR suppressor domain, respond to a large number of stress conditions and are considered abiotic stress markers [41]. Finally, *ERF2* (*ETHYLENE RESPONSE FACTOR 2*) encodes an APETALA2 (AP2)/ERF TF, whose expression is altered after MeJA treatment, that has been shown to positively regulate JA-responsive defense genes and enhance JA-dependent inhibition of root elongation [42]. Besides TFs, among genes downregulated in *athb1,* we also found genes involved in JA and SA synthesis. For example, *LOX4* encodes one of the four 13-lipoxygenases (13-LOXs) that produce JA precursors in aerial tissues in *Arabidopsis* [43]. *ENHANCED DISEASE SUSCEPTIBILITY 1* (*EDS1*) encodes an SA-induced transcriptional cofactor that positively regulates the SA biosynthesis gene *ICS1* (*ISOCHORISMATE SYNTHASE1*), forming a positive feedback loop that enhances SA response [44]. *ENHANCED DISEASE SUSCEPTIBILITY 5* (*EDS5),* whose expression is regulated by SA and JA, encodes a transporter necessary for the export of isochorismate from chloroplasts to the cytoplasm [45]. In addition, other genes are involved in JA and SA signaling like *GRX480* (*GLUTAREDOXIN480*, also known as *ROXY19*) [46], *AT2G32140 (TIR-X)*, encoding a protein with a TIR (Toll/interleukin-1 receptor) domain [47], the JA and wound response marker *TYROSINE AMINOTRANSFERASE 3* (*TAT3)* [48], the SA induced aspartyl protease encoded by *APOPLASTIC, EDS1-DEPENDENT 1* (*AED1*) [49], the WRKY70 target *LATE UPREGULATED IN RESPONSE TO Hyaloperonospora parasitica 1 (LURP1)* [50], the ubiquitin 26S proteasome system substrate adaptor protein *F-BOX STRESS-INDUCED 1* (*FBS1*) [51] and the *MITOGEN-ACTIVATED PROTEIN KINASE KINASE 4* (*MKK4*) required for the SA-promoted leaf senescence [52]. Finally, several genes encoding CALMODULIN-LIKE (CMLs, *CML10*, *CML38*, *CML44*, *CML46*) involved in Ca^2+^ sensing [53] and *CALCIUM-DEPENDENT PROTEIN KINASE 29* (*CPK29*) that interprets Ca^2+^ signals from internal and external triggers [54] are also found. In particular, CML38 is acutely induced early in hypoxia and is a signaling hub during low oxygen stress in *A. thaliana* [53]. Amongst the genes whose expression is increased in *athb1,* we found *FLOWERING LOCUS C (FLC)* and *1-AMINO-CYCLOPROPANE-1-CARBOXYLIC ACID (ACC) OXIDASE 3* (*ACO3)*. *FLC* encodes a MADS box TF that is a central flowering regulator in *Arabidopsis* integrating vernalization and autonomous pathways [55]. *ACO3* encodes an enzymatic protein that converts ACC to ethylene, particularly involved in ethylene biosynthesis in the root [56].

To further explore the interactions between *ATHB1* and JA signaling, we compared the 119 differentially regulated genes in *athb1* relative to WT under basal conditions with the two datasets of differentially regulated genes in the WT 1 h and 4 h after MeJA application. We found that expression of about 50% of the genes differentially expressed in *athb1* with respect to WT respond to MeJA treatment, and about 20% of them show differential expression at both time points (Figure 3C). Genes induced and repressed by MeJA treatment are almost equally present and include genes encoding transcription factors (*JAZ1*, *JAZ5*, *NAC36*, *WRKY40*, *ERF2*, *ZAT10*, *ZAT12*) and proteins involved in JA response (*LOX4*, *TAT3*, *TIR-X*, *GRX480*). Hierarchical clustering of DEGs profiles in WT and *athb1* after MeJA application confirms that the transcriptional response to the hormone is not substantially affected in the mutant, as previously shown by the analysis of the whole transcriptome (Table 1). A set of 15 genes differentially regulated in *athb1* relative to WT (2 upregulated and 13 downregulated) was chosen to validate the results of the microarray by RT-qPCR analysis. The results, shown in Table 2, indicate a good correlation between log_2_FC values obtained using RT-qPCR and RNA microarray, demonstrating data reliability.

ATHB1 binds the pseudo palindromic sequence CAAT(A/T)ATTG (BS-I site) in vitro [57,58,59] and in transient expression experiments [15]. Therefore, we investigated which of the genes whose transcript level was altered in the *athb1* mutant background have this target sequence in the promoter region by analyzing their 5′ upstream sequences deposited in *Arabidopsis* databases. Considering that the lateral positions may have greater variability [57], we first scanned the promoter sequences of the differentially expressed genes for the presence of BS-I site core AAT(A/T)ATT and found that this motif is present at least once in 87% of the DEGs (Supplementary Table S1). Among these, four genes exhibited the full, nine bp-long ATHB1 binding site in the 3000 bp preceding the transcription start site (Supplementary Table S1).

## 4. Discussion

Plant growth exhibits remarkable plasticity, enabling adaptation of the intrinsic developmental program to varying and often challenging environmental conditions. This flexibility is largely mediated by the intricate interaction between hormonal signaling pathways and transcriptional regulation. Among the various TF families involved, the HD-ZIP I family, to which ATHB1 belongs, stands out for its potential in crop improvement, as some of these TFs have been shown to influence key developmental processes and to respond to hormone signals and biotic and abiotic stresses [7,13,60,61,62]. In particular, data in the literature indicate that ATHB1 controls hypocotyl elongation under short-day regimes [17] and affects leaf cell expansion [15] and leaf margin serration through direct repression of miR164 and regulation of *CUC2* expression [16]. Furthermore, *ATHB1* expression was found to be regulated negatively by ABA, salt and cold stress [7], positively by ethylene [18] and oppositely by drought in plants cultured in vitro [7], or grown in soil [12]. In addition, mining of the BAR repository for expression data of HD-Zip members in abiotic stress experiments highlighted that, in roots, *ATHB1* was induced by osmotic, genotoxic, UV-B, and high-temperature conditions, whereas it was slightly repressed by sodium chloride [60]. In this study, we demonstrated that *ATHB1* is also induced by other stresses, namely flooding and wounding. Moreover, we found that, in leaves, *ATHB1* expression is affected by the ethylene signal transduction pathway, being reduced in *ein-2* and *etr-1* mutants. However, an age-dependent increase of the *ATHB1* transcript level is still observed in these mutants, suggesting that the gene is also under developmental control. Several studies have indicated that ethylene can act in a coordinated or antagonistic manner with JA to regulate plant defense against stress, and cross-regulation of the two signaling pathways can occur through convergence on key TFs that confer abiotic stress tolerance, such as ethylene response factors (ERFs) [4,31,32]. Integration of the JA and ethylene signaling pathways has also been reported for an HD-ZIP I TF through the functional analysis of transgenic *Arabidopsis* and maize plants constitutively expressing the sunflower *HaHB4* gene [63]. In addition, analysis of the genes resulting from the transcriptome comparison of *Arabidopsis thaliana* plants over-expressing HD-Zip I proteins linked to water deficit tolerance have pointed out hormone responses (jasmonic acid, salicylic acid, ethylene, gibberellin and ABA) and biotic and abiotic stresses (defense response and water deprivation) among the enriched GO terms and the ethylene-related pathway as the most relevant in co-expression analysis [12]. Although JA signaling contributes to stress tolerance, the application of MeJA during vegetative development generally impairs plant growth [48,64,65]. By growing plants in the presence of MeJA, we found that growth inhibition is influenced by the level of *ATHB1* expression, with sensitivity decreasing in *athb1* plants and increasing in ATHB1 OE plants compared to WT plants. Despite this physiological evidence, a comprehensive gene expression analysis using microarray revealed that the transcriptional response is not significantly affected by MEJA treatment in the *athb1* mutant. In fact, only seven genes were found to be differentially expressed in the mutant compared to WT plants after 4 h of MeJA treatment, and none after 1 h. Notably, we observed that this differential regulation was likely due to different baseline expression levels in untreated WT and *athb1* plants rather than a distinct response of the two genotypes to hormone application. Analysis of the untreated samples allowed us to identify 119 high-confidence DEGs that are potentially regulated by ATHB1. The majority (79) of these DEGs are downregulated, while only about one-third (40) are upregulated in the mutant background. Interestingly, enrichment analysis reveals that GO terms associated with JA signaling and response are significantly enriched among the downregulated genes. In addition, about half of the DEGs respond to MeJA treatment in both WT and *athb1* seedlings, suggesting that ATHB1 influences the basal expression of genes affected by the JA signaling, although it is not essential for their response following MeJA application. Thus, the reduced expression levels of transcription factors (JAZ1, JAZ5, WRKY40, ERF2, ZAT10) and proteins (LOX4, TAT3, TIR-X, GRX480) involved in JA signaling in untreated plants may explain the decreased sensitivity of *athb1* plants to MeJA application.

Interestingly, among the downregulated genes in the *athb1* mutant, there are also important regulators of the SA-mediated response. For instance, *NAC90* is part of a “NAC troika” shown to regulate genes involved in SA synthesis and downstream TFs of SA signaling, including *WRKY40* and *WRKY70,* whose expression is also affected in *athb1* [66]. Specifically, *NAC90* has been identified as a negative regulator of SA-mediated leaf senescence, directly binding and suppressing promoters of target genes, including *EDS5* [66], which is also downregulated in *athb1*. This leads to a reduction of SA accumulation at the pre-senescent stage [66]. Another “NAC triad”, including both *NAC90* and *NAC36*, was recently shown to negatively regulate plant immunity by directly repressing expression of N-hydroxy pipecolic acid and SA biosynthetic genes [67]. As transcription of both *NAC90* and *NAC36* is induced by SA, a negative feedback loop is established upon pathogen infections to prevent excessive SA accumulation, early senescence and growth inhibition [67]. *WRKY40* and *WRKY70* encode proteins belonging to the WRKY family, the largest and most extensively studied group of plant TFs involved in biotic and abiotic stress responses and adaptation [68]. It is important to emphasize that WRKY TFs often act in concert with proteins of the VQ motif-containing family of plant-specific transcriptional regulators [69] and one member of this group, *VQ10*, is also found to be downregulated in the *athb1* mutant. Notably, *WRKY70* expression is responsive to reactive oxygen species and shows interesting similarities with *ATHB1* regarding tissue specificity, being present in both stigmatic papillae and the flower abscission zone [70], as well as in leaves, where its level gradually increases with leaf senescence [70,71]. Finally, it is worth mentioning *EDS1* and *GRX480,* encoding two transcriptional cofactors induced by SA and acting negatively on promoters of JA-responsive genes, thus participating in SA/JA crosstalk [44,46].

**Figure 4:**
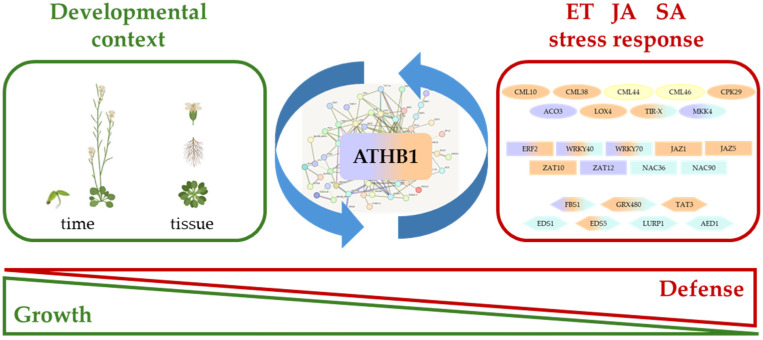
The model illustrates the interaction of *ATHB1* with hormone-dependent transcriptional regulatory networks, revealed by this study, and the proposed role of *ATHB1* in the integration of developmental and stress signals affecting the growth-defense balance under unfavorable environmental conditions. DEGs related to hormone signaling are colored in lilac (ethylene, ET), orange (JA) or cyan (SA). Proteins involved in hormone synthesis or signaling are represented as ovals, TFs as rectangles and effector proteins as hexagons.

Overall, our data suggest that *ATHB1*, whose expression is regulated during plant growth and development, as well as by ethylene and JA, may interact with transcriptional regulatory networks mediating ethylene-, JA- and SA-dependent stress responses (Figure 4). Depending on the cell type, developmental stage, and growth conditions, ATHB1 activity—also regulated at post-transcriptional levels through CpuORF33 [21]—may influence the production of molecules involved in stress signal perception and transduction by modulating stress-responsive TFs and their targets. This regulatory network may establish a specific basal physiological state, which, in turn, affects the amplitude of the defense response and the organism’s ability to adapt and survive under unfavorable and stressful conditions.

## 5. Conclusions

The data presented in this study, along with findings from the literature, suggest that *ATHB1* functionally interacts with key regulators of plant growth and development (e.g., light, hormones) as well as abiotic and biotic stress responses. Further studies are needed to explore how the interplay between *ATHB1* and signaling pathways can be exploited, including in relevant crops, to enhance plant growth and productivity under various stress conditions. This could help maximize the activation of acclimation and defense pathways while minimizing negative pleiotropic effects on growth and yield.

## Figures and Tables

**Table 1:** List of genes differentially expressed in microarray in the comparison (*athb1* MeJA/mock)—(WT MeJA/mock) 4 h after MeJA application and expression of the selected genes in untreated *athb1* respect to WT (1 h and 4 h mock).

AGI Code	Gene	log_2_ FC	log_2_ FC		Gene Annotation
		(*athb1* 4 h MeJA/mock)–(WT 4 h MeJA/Mock)	*athb1*/WT Mock 1 h	*athb1*/WT Mock 4 h	
AT1G27730	STZ/ZAT10	1.51	−1.06	−1.41	SALT TOLERANCE ZINC FINGER; nucleic acid binding; transcription repressor; zinc ion binding; response to oxidative stress, response to wounding, response to salt stress
AT1G76650	CML38	1.36	−0.77 *	−1.6	CALMODULIN-LIKE 38; calcium-binding EF hand family protein; response to wounding; positive regulation of brassinosteroid mediated signaling pathway, response to hypoxia
AT2G17040	NAC036	1.27	−1.33	−1.46	NAC DOMAIN CONTAINING PROTEIN 36; transcription factor; involved in leaf and inflorescence stem morphogenesis
AT4G39030	EDS5	1.14	−0.65	−1.51	ENHANCED DISEASE SUSCEPTIBILITY 5; orphan multidrug and toxin extrusion transporter; essential component of salicylic acid-dependent signaling
AT1G17380	JAZ5	1.12	−0.7	−1.05	JASMONATE-ZIM-DOMAIN PROTEIN 5; regulation of jasmonic acid mediated signaling pathway; involved in response to wounding
AT2G39030	NATA1	1.12	−0.22 *	−0.21 *	N-ACETYLTRANSFERASE ACTIVITY 1; ornithine metabolic process, polyamine acetylation; response to jasmonic acid
AT1G78410	VQ10	1.1	−0.91	−1.55	VQ MOTIF-CONTAINING PROTEIN 10; response to oxidative stress; protein binding, interaction with WRKY transcription factors

* Values not statistically significative.

**Table 2:** RT-qPCR validation of microarray results for 15 genes differentially expressed in athb1 seedlings compared with WT (mock treated 4 h).

AGI Code	Gene	Annotation	Microarray (log_2_ FC)	RT-qPCR Validation * (log_2_ FC)
AT5G10140	FLC	FLOWERING LOCUS C; MADS-box Transcription Factor; specific transcriptional repressor; regulation of flower development, response to abiotic stimulus.	1.71	2.78 ± 0.18
AT1G12010	ACO3	1-Amino-Cyclopropane-1-Carboxylic Acid Oxidase 3; ethylene biosynthesis.	0.80	1.27 ± 0.36
AT5G47220	ERF2	Ethylene Response Factor-2; ERF/AP2 transcription factor family; transcriptional activator; ethylene-activated signaling pathway; jasmonic acid mediated signaling pathway.	−0.74	−1.01 ± 0.61
AT2G39030	NATA1	N-ACETYLTRANSFERASE ACTIVITY 1; ornithine metabolic process, polyamine acetylation; response to jasmonic acid.	−0.21 **	−0.72 ± 1.08
AT1G17380	JAZ5	Jasmonate-ZIM-domain Protein 5; regulation of jasmonic acid mediated signaling pathway; response to wounding.	−1.05	−1.10 ± 0.41
AT3G48090	EDS1	Enhanced Disease Susceptibility 1; component of R gene-mediated disease resistance; acts redundantly with salicylic acid to regulate resistance gene-mediated signaling; lipase; signal transducer.	−1.10	−1.04 ± 0.05
AT4G21090	MFDX2	Mitochondrial Ferredoxin 2; metal ion binding.	−1.29	−1.00 ± 0.39
AT1G27730	ZAT10	STZ (salt tolerance zinc finger); nucleic acid binding; transcriptional repressor involved in abiotic stress responses; probably involved in jasmonate (JA) early signaling response; response to oxidative stress, response to wounding, response to salt stress; zinc ion binding.	−1.41	−1.57 ± 0.14
AT2G17040	NAC36	Arabidopsis NAC domain containing protein 36; transcription factor; involved in leaf and inflorescence stem morphogenesis.	−1.46	−1.39 ± 0.05
AT1G80840	WRKY40	Pathogen-induced transcription factor; binds W-box sequences in vitro; regulation of defense response; response to salicylic acid; response to wounding.	−1.50	−1.83 ± 0.59
AT4G39030	EDS5	Enhanced Disease Susceptibility 5; multidrug and toxin extrusion transporter; component of salicylic acid-dependent signaling.	−1.51	−1.68 ± 0.08
AT1G78410	VQ10	VQ motif-containing protein, response to oxidative stress; protein binding, interaction with WRKY transcription factors.	−1.55	−2.26 ± 0.48
AT1G76650	CML38	CALMODULIN-LIKE 38; calcium-binding EF hand family protein; response to wounding; positive regulation of brassinosteroid mediated signaling pathway, response to hypoxia.	−1.6	−1.76 ± 0.26
AT5G22380	NAC90	NAC domain containing protein 90; Transcription Factor.	−1.70	−4.61 ± 0.94
AT3G56400	WRKY70	Transcription factor involved in senescence, biotic and abiotic stress responses by modulating various phytohormones signaling pathways; function as activator of SA-dependent defence genes and a repressor of JA-regulated genes; ethylene signaling pathway; BR signaling pathway.	−1.97	−2.49 ± 0.59

* Values are mean ± SD of relative quantification from three independent experiments, each with three technical replicates. ACTIN2 was used as reference gene for normalization. ** Value not statistically significative.

## Data Availability

All data and materials presented in this paper are available on request from the corresponding author. Data supporting the reported results are available in a publicly accessible repository as NASCarrays experiment no. 572, release date 24 May 2012 and can be found at The BAR (http://bar.utoronto.ca/NASCArrays/index.php?ExpID=572; accessed on 9 September 2025).

## Supplementary Materials

The following supporting information can be downloaded at: https://www.mdpi.com/article/10.3390/cells14181456/s1. Figure S1: Histochemical localization of GUS activity in transgenic *ATHB1*::GUS seedlings; Figure S2: Phenotype of *athb1-3* mutant and ATHB1 OE plants [16]; Table S1: List of differentially expressed genes in *athb1* with respect to WT seedlings; Table S2: List of GO Biological Process (BP) category; Table S3: List of primers.
