# Supplementary material for: ATHB1 Interacts with Hormone-Related Gene Regulatory Networks Involved in Biotic and Abiotic Stress Responses in Arabidopsis
Source: Cells. 2025 Sep 17;14(18):1456. doi: 10.3390/cells14181456 (PMC12468107; doi:10.3390/cells14181456)
Supplement: Supplementary file 1 [file cells-14-01456-s001.zip › proofreading_Forte et al_Supplementary Figures_S1_S2.pdf]

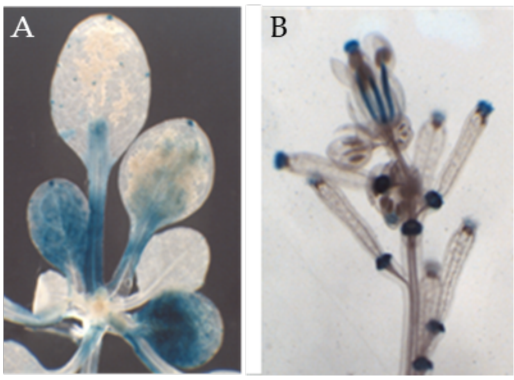

Supplementary Figure S1. Histochemical localization of GUS activity in transgenic *ATHB1*::GUS seedlings (**A**) rosette leaves of 3-week-old plants and (**B**) inflorescence of 6-week-old plants.

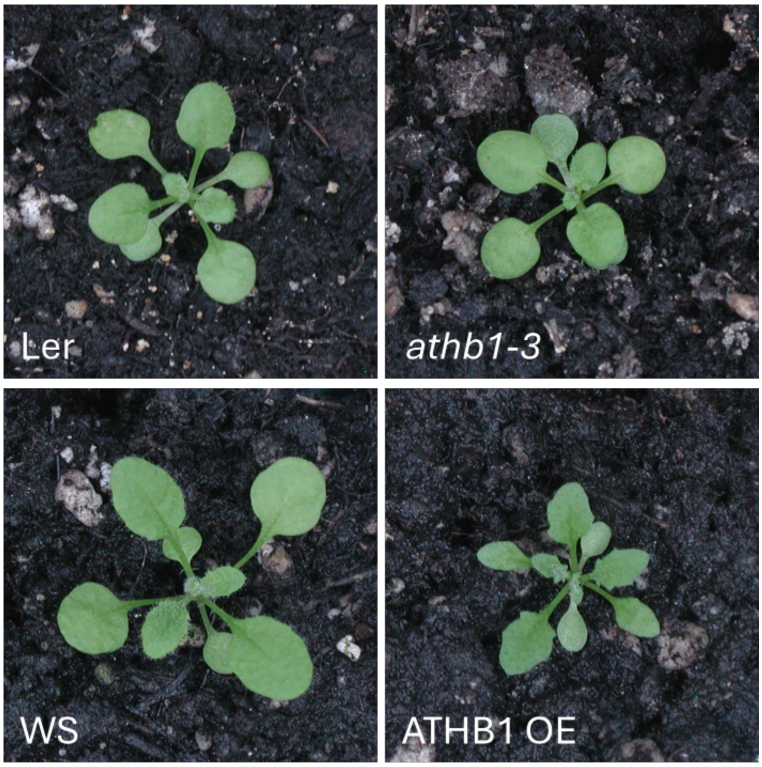

Supplementary Figure S2. Phenotype of *athb1-3* mutant and ATHB1 overexpressing (ATHB1 OE) plants and of their respective WT (left panels). *athb1-3* shows overall size, leaf and flower morphology similar to WT while ATHB1 OE plants display increased leaf margin serration as already reported [16].
