# Supplementary material for: ATHB1 Interacts with Hormone-Related Gene Regulatory Networks Involved in Biotic and Abiotic Stress Responses in Arabidopsis
Source: Cells. 2025 Sep 17;14(18):1456. doi: 10.3390/cells14181456 (PMC12468107; doi:10.3390/cells14181456)
Supplement: Supplementary file 1 [file cells-14-01456-s001.zip › proofreading_Forte et al_Supplementary Table S3.pdf]

Supplementary Table S3. Sequences of primers used for gene expression analysis by RT-qPCR

AGI code	Gene name	Forward primer	Reverse primer
AT1G12010	*ACO3*	5'-TAGGGCTCACACAGATGCAG-3'	5'-GGAACATCAACCCAATCACC-3'
AT1G17380	*JAZ5*	5'-TGCTAAACGGAAAGACAGAGC-3'	5'-TGTCCTGCCTCTAGTGGTTGA-3'
AT1G27730	*ZAT10/STZ*	5'-GGACAAAGGGTAAGCGATCTAA-3'	5'-AGAAGCATGAGGCAAAAAGC-3'
AT1G76650	*CML38*	5'-CATGGATGCAAACAGAGACG-3'	5'-GCAGCTACGGCTTCTTCATC-3'
AT1G78410	*VQ10*	5'-GGCGGTAGTAAAATCGGTGA-3'	5'-CTATCGAACTCCGTCGTCGT-3'
AT1G80840	*WRKY40*	5'-CTTGACTGTGCCGGTGACTA-3'	5'-TCTGAACTTGGGGAAAATCG-3'
AT2G17040	*NAC36*	5'-TGATGAACGAGTTTCGGATG-3'	5'-TCTGCCCTTTGCTCCAATAC-3'
AT2G39030	*NATA1*	5'-GTGGCTGGATTCGTTCTGTT-3'	5'-GCGGTCAGTAGCAGTTTTCC-3'
AT3G01470	*ATHB1*	5′-TCCGAGGTTACTTCCCTGACCGAA-3′	5′-GGCACTTGACCAGGTGGTTCATTA-3′
A3G18780	*ACT2*	5'-GACCAGCTCTTCCATCGAGAA-3'	5'-CAAACGAGGGCTGGAACAAG-3'
AT3G48090	*EDS1*	5'-GAAGACACAGGGCCGTACAT-3'	5'-ATCATTCCGTTTGGCTTCAG-3'
AT3G56400	*WRKY70*	5'-GAACCCATCTCCTCCTCCTC-3'	5'-TATCGCCGGAATCTTCAAAC-3'
AT4G21090	*MFDX2*	5′-TCATCGCAAAACCAGAGCTAG-3′	5′-GAACAAACCCATCAACCGC-3′
AT4G39030	*EDS5*	5'-CGAACTCGCTGCTCTTGG-3'	5'-GCAACCATATTGGATGTAGCC-3'
AT5G10140	*FLC*	5'-TGTGGATAGCAAGCTTGTGG-3'	5'-GAGAGGGCAGTCTCAAGGTG-3'
AT5G22380	*NAC90*	5'-CTACGAAACCAGCTCGAAGG-3'	5'-AGAACCATTGCTCAGCGTCT-3'
AT5G47220	*ERF2*	5'-GGAGGTTTGCCATTGAAAGA-3'	5'-GCCGGAAAATCAAAAAGACA-3'
